# Genetic Structure of the Norwegian *Parastagonospora nodorum* Population

**DOI:** 10.3389/fmicb.2020.01280

**Published:** 2020-06-16

**Authors:** Min Lin, Andrea Ficke, James Cockram, Morten Lillemo

**Affiliations:** ^1^Department of Plant Sciences, Faculty of Biosciences, Norwegian University of Life Sciences (NMBU), Ås, Norway; ^2^Division of Biotechnology and Plant Health, Norwegian Institute of Bioeconomy Research (NIBIO), Ås, Norway; ^3^John Bingham Laboratory, NIAB, Cambridge, United Kingdom

**Keywords:** *Parastagonospora nodorum*, Septoria nodorum blotch, genetic variation, population genetics, wheat, simple sequence repeat markers, leaf blotch diseases

## Abstract

The necrotrophic fungal pathogen *Parastagonospora nodorum* causes Septoria nodorum blotch (SNB), which is one of the dominating leaf blotch diseases of wheat in Norway. A total of 165 *P. nodorum* isolates were collected from three wheat growing regions in Norway from 2015 to 2017. These isolates, as well as nine isolates from other countries, were analyzed for genetic variation using 20 simple sequence repeat (SSR) markers. Genetic analysis of the isolate collection indicated that the *P. nodorum* pathogen population infecting Norwegian spring and winter wheat underwent regular sexual reproduction and exhibited a high level of genetic diversity, with no genetic subdivisions between sampled locations, years or host cultivars. A high frequency of the presence of necrotrophic effector (NE) gene *SnToxA* was found in Norwegian *P. nodorum* isolates compared to other parts of Europe, and we hypothesize that the *SnToxA* gene is the major virulence factor among the three known *P. nodorum* NE genes (*SnToxA*, *SnTox1*, and *SnTox3*) in the Norwegian pathogen population. While the importance of SNB has declined in much of Europe, Norway has remained as a *P. nodorum* hotspot, likely due at least in part to local adaptation of the pathogen population to ToxA sensitive Norwegian spring wheat cultivars.

## Introduction

*Parastagonospora nodorum* (syn. *Phaeosphaeria nodorum*, *Septoria nodorum*, *Stagonospora nodorum*, or *Leptosphaeria nodorum*) is one the most devastating necrotrophic fungal pathogens of wheat (*Triticum aestivum*), causing septoria nodorum blotch (SNB) on wheat leaves and glume blotch on glumes (Wicki et al., 1999). The yield loss caused by SNB can reach 50% when susceptible cultivars are grown under weather conditions conducive for *P. nodorum* (Eyal, 1981). Currently, no commercially available cultivar has shown complete resistance to SNB, so tillage, crop rotation and chemical control are still the most effective disease management practices used.

Epidemics of *P. nodorum* have been reported in all six continents where wheat is grown (Leath et al., 1993; Ficke et al., 2018a). SNB causes necrotic lesions in wheat leaves, with similar symptoms observed for other leaf blotch diseases of wheat, such as *Septoria tritici* blotch (STB, caused by *Zymoseptoria tritici*) and tan spot (caused by *Pyrenophora tritici-repentis*). Growth of these three pathogens is favored by warm and humid weather conditions, with dispersal of asexual spores mediated by rain splash and/or wind (Morrall and Howard, 1975; Bearchell et al., 2005) and often cause co-infections on wheat in Europe (Jalli et al., 2011; Ficke et al., 2018b). In Norway, *P. nodorum* was assumed to have arrived with the import of wheat seeds, as the oldest documented epiphytotics was in 1894, Aker in Oslo (Jørstad, 1967). It has been a pathogen with economic importance along with the rise in the wheat production in Norway since the 1970s (Jørstad, 1967; Ruud and Lillemo, 2018). Although systematic *P. nodorum* incidence data from all European countries over the last 20 years is not available, fewer *P. nodorum* epidemics have been observed compared to *Z. tritici* in western Europe since the 1980s (Wiik, 2009). In the United Kingdom for example, data from 1844 to 2003 indicates that *P. nodorum* was the predominating pathogen on wheat until 1980s, after which its dominance was replaced by *Z. tritici* (Bearchell et al., 2005; Shaw et al., 2008). This change in pathogen dominance was correlated with the reduction of sulfur pollution, although other explanations were proposed earlier such as differences in cultivar resistance and response to fungicide application (Shaw et al., 2008). However, reduction of sulfur in the atmosphere does not explain why *P. nodorum* still dominates in western Australia (Oliver et al., 2012) and Norway. Indeed in Norway *P. nodorum* is still the dominating leaf blotch pathogen of wheat, and sulfur pollution has not been reported to be higher than in any other of the European countries in which *Z. tritici* has come to dominate the leaf blotch complex in wheat.

As a model organism for necrotrophic fungal pathogens, *P. nodorum* is known to produce necrotrophic effectors (NEs), which interact with wheat effector sensitivity loci, causing programmed cell death in order to accelerate infections (Friesen et al., 2007; Oliver et al., 2012). So far, eight *P. nodorum* NEs have been characterized, (reviewed by Ruud and Lillemo, 2018) and three NE coding genes have been cloned: *SnToxA*, *SnTox1*, and *SnTox3* (Liu et al., 2006, 2009, 2012). ToxA encoded by the *P. nodorum* gene *SnToxA*, was first characterized as a virulence factor of *P. tritici-repentis* (Tomas et al., 1990). Subsequently, it was shown that the *SnToxA* gene likely originated from *P. nodorum* and was passed on to *P. tritici-repentis* during a recent horizontal gene transfer (Friesen et al., 2006). Horizontal gene transfer of *P. nodorum SnToxA* into another wheat pathogen *Bipolaris sorokiniana*, has recently been reported in natural populations in Australia (McDonald et al., 2018) and the United States (Friesen et al., 2018). Reducing the growing area of ToxA sensitive cultivars or eliminating the wheat ToxA susceptibility locus *Tsn1* from breeding programs might reduce yield loss due to leaf blotch diseases substantially, since *SnToxA* is a virulence factor of three different wheat pathogens.

Population genetics studies of *P. nodorum* have previously been carried out at national to global scales using either restriction fragment length polymorphism (RFLP) probes or simple sequence repeat (SSR) markers, and high genetic variability within *P. nodorum* populations were observed (Keller et al., 1997; Murphy et al., 2000; Stukenbrock et al., 2006; Blixt et al., 2008). The reason why Norway is still one of the few countries in Europe where SNB remains the dominating leaf blotch disease of wheat remains unknown. One explanation could include the highly specialized host pathogen relationship based on necrotrophic effectors and their corresponding susceptibility genes in the wheat varieties grown in Norway. However, the genetic structure of the *P. nodorum* pathogen population in Norway and genotypic analysis of their NE genes has not been characterized to date, as Norwegian isolates were not included in the previously published global *P. nodorum* genetic studies (Stukenbrock et al., 2006; McDonald et al., 2013).

The purpose of the current study was to establish a Norwegian *P. nodorum* isolate collection, and to study their genetic structure and NE gene allele frequencies and to compare these genetic datasets with isolates from other countries. Specifically, we (1) established a collection of 165 Norwegian *P. nodorum* isolates, (2) genotyped the collection using 20 SSRs, (3) calculated both regional and nation-wide *P. nodorum SnTox* gene (*SnToxA*, *SnTox1*, and *SnTox3*) frequencies (4) compared the *P. nodorum* isolates collected from winter wheat and spring wheat, (5) investigated the relationship between *SnTox* gene frequencies and the cultivars where isolates were collected from, (6) assessed the multi-effector genotype distribution and the correlation with the corresponding cultivar NE sensitivities.

## Materials and Methods

### Sampling

**FIGURE 1:**
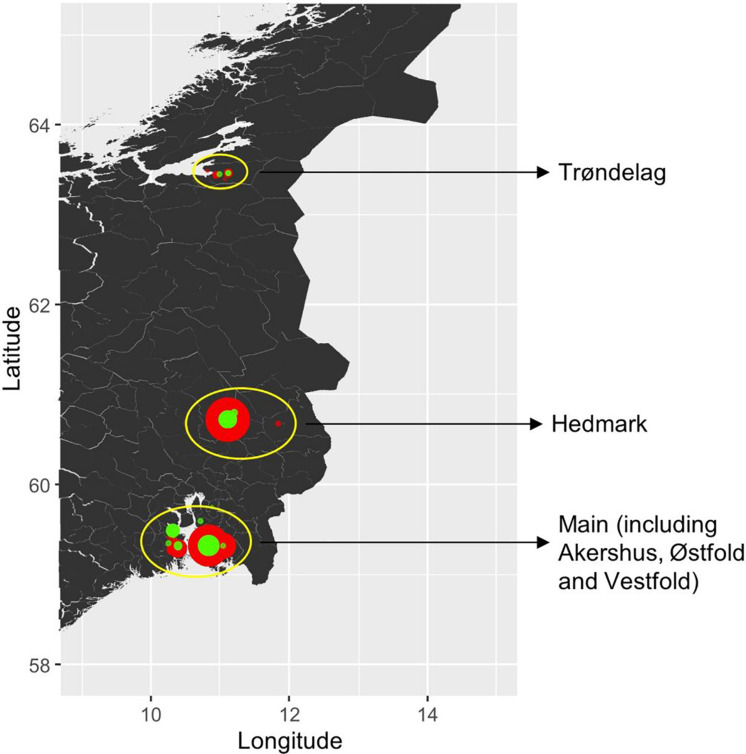
Sampling locations of *P. nodorum* isolates in Norway. The wheat cultivar types from which isolates were collected from are coded by color (red: winter cultivars; green: spring cultivars), and the size of each dot indicates the sample size.

*P. nodorum* isolates were collected from 23 fields in five wheat growing counties in Norway (Akershus, Østfold, Vestfold, Hedmark, and Trøndelag). As Akershus, Østfold and Vestfold counties are geographically close to each other and have similar climate, these three regions were grouped as a single large region in this study (Figure 1). Field sampling was undertaken in 2015, 2016, and 2017. Roughly 30 wheat leaves were collected per cultivar per wheat field, which was naturally infected by *P. nodorum*. Leaf samples were collected from a total of 13 cultivars, dried and kept at room temperature. Only one single spore isolate was collected per leaf. In 2015, samples were collected only from winter wheat, while in 2016 and 2017 samples were collected from both winter and spring wheat cultivars. Except Jantarka, all cultivars have been tested for sensitivity to three known *P. nodorum* effectors (ToxA, Tox1, and Tox3) (Ruud et al., 2018). Two isolates (Isolate ID: 201865 and 201982) were collected from wheat leaf samples sent by EffectaWheat project collaborators in 2016 from Germany and Denmark, respectively. One isolate (ID: 202580) was received from our EffectaWheat project collaborator in the United Kingdom. An additional set of six isolates from Switzerland (Sn99CH1A7a), United States (Sn6 and Sn79-1087), Mexico (CIMFU460-SN1 and CIMFU463-SN4), and Australia (SN15) were also included in this study.

### Fungal Material for DNA Extraction

Isolates were grown on petri dishes containing Potato Dextrose Agar (PDA) for 14 days at 20°C in darkness to promote mycelium growth. DNA was extracted using the DNEasy plant DNA Extraction Kit (Qiagen, Hilden, Germany) from fungal biomass which was scraped from the surface of each petri dish.

### Determination of Mating Type and *SnTox* Gene Profiles

To identify the mating type idiomorphs of each isolate, the mating type primers described by Bennett et al. (2003) were utilized for multiplex polymerase chain reaction (PCR), as described by Sommerhalder et al. (2006). For mating type MAT1-1, a 360 bp fragment was amplified while for MAT1-2, a 510 bp fragment was amplified. The MAT1-1 to MAT1-2 idiomorphs’ ratio was tested to determine whether the deviation was significantly different from the 1:1 ratio by Chi-square test. Genotyping of *Actin* and *SnTox* genes (*SnToxA*, *SnTox1*, and *SnTox3*) was performed by PCR as described by Gao et al. (2015). The Chi-squared test for given probabilities was carried out to compare *SnTox* gene allele frequency with the global *P. nodorum* collection published by McDonald et al. (2013). Pearson’s Chi-squared tests were used to compare *SnTox* gene allele frequencies between locations, cultivars, wheat types and another Norwegian *P. nodorum* isolate collection from spring wheat (Ruud et al., 2018).

### SSR Analysis

Three expressed sequence tag (EST) derived SSR loci SNOD1, SNOD3, SNOD5, one minisatellite locus SNOD8 (Stukenbrock et al., 2005) and 16 newly developed SSR loci (Supplementary Table S1) were used for genotyping. The new SSR markers were designed by Dr. Patrick C. Brunner at ETH Zurich based on the reference genome SN15 and alignments with genome sequences of 164 global strains of *P. nodorum* (Pereira et al., 2019). PCR was carried out with M13 tailed (Schuelke, 2000) fluorescent labeled primers (Supplementary Table S1), PCR products were separated by capillary electrophoresis using an ABI3730 Gene Analyzer (Applied Biosciences) and a GeneScan 500 LIZ dye Size Standard from Applied Biosystems (Life Technologies), and the resulting data analyzed using Software: GeneMapper v.5 (Applied Biosystems).

### Population Genetic Analyses

The genotype accumulation curve was calculated in R Studio Version 1.1.442 (R Studio Team, 2015) using the function genotype_curve implemented in the R package “poppr” (Kamvar et al., 2014) in order to determine the number of loci required to discriminate individuals in a population. A UPGMA (unweighted pair group method with arithmetic mean) tree was created with non-parametric bootstrapping (*n* = 100) using the function bruvo.boot/poppr, with genetic distance between individuals estimated by Bruvo’s distance (Bruvo et al., 2004) and a distance matrix generated by the UPGMA hierarchical clustering method. Genotype diversity information, including allele frequency, Simpson’s index (λ) (Simpson, 1949) unbiased gene diversity H_exp_ (Nei, 1973) and evenness of each SSR locus, were calculated using the poppr package (Kamvar et al., 2014). In addition, the index of association (I_A_) (Brown et al., 1980) the standard index of association (r_d_), and corresponding *p*-values for I_A_ and r_d_ were calculated using 1000 permutations with the ‘poppr‘ package (Kamvar et al., 2014) to test the null hypothesis of linkage equilibrium of SSR loci due to random mating. The analysis of molecular variance (AMOVA) was also carried out by function implemented in the ‘poppr‘ package.

Population structure analysis of the Norwegian *P. nodorum* isolates was done using three different approaches. (1) Using STRUCTURE v. 2.3.4 (Pritchard et al., 2000; Falush et al., 2003). The number of genetic populations were tested from *K* = 1 to *K* = 10, and each K was iterated 10 times. The following parameters were used for structure analysis: admixed model using sampling location as prior, initial burn-in period of 30,000 with 10^6^ additional cycles. The best value for K was estimated based on the deltaK approach (Evanno et al., 2005) implemented in Structure Harvester (Earl and vonHoldt, 2011). (2) Estimating SSR variation between isolates using principal component analysis (PCA) implemented in the R packages ade4 and adegenet (Dray and Dufour, 2007; Jombart, 2008). (3) Estimating the optimalnumber of K using the “snapclust” function implemented in adegenet/R (Jombart, 2008; Beugin et al., 2018) which combined both geometric and fast likelihood optimization.

## Results

### Sampling

A total of 165 *P. nodorum* isolates were collected in Norway over 3 years, from 2015 to 2017. Sample site information is listed in Supplementary Table S2, including geographical origin, year of collection and from which wheat cultivar leaf material was collected. In summary, 48 isolates were collected in 2015, 74 isolates in 2016 and 43 isolates in 2017. Among those, 31% of the whole collection were isolated from spring wheat cultivars while the remaining 69% were isolated from winter wheat cultivars. From a geographical perspective, 92 isolates were collected from the main wheat growing region including Akershus, Østfold, and Vestfold (southeast of Norway), 53 isolates were from Hedmark (inland Norway) and 20 isolates were from Trøndelag (central Norway) (Figure 1).

### Genetic Diversity of Norwegian *P. nodorum* Isolates

All 20 SSR loci were amplified successfully across all 174 *P. nodorum* isolates, with less than 5% missing data for each locus. Based on these SSR profiles, the genotype-accumulation curve showed that the whole *P. nodorum* collection (including the nine international isolates) had 173 multilocus genotypes (MLG) (Supplementary Figure S1). The minimum number of loci required to distinguish all individuals in this collection was 7 (Supplementary Figure S1). Clone-corrections based on SSR profiles indicated that no clonal isolates were detected in any single region, however, isolate 202552 collected from Hedmark and isolate 202522 collected from Trøndelag showed the same SSR profile (Table 1). Therefore, only one isolate with this MLG was kept for the following clone-corrected analyses. The genetic diversity H_exp_ of the 16 new SSR markers ranged from 0.47 to 0.93 which was comparable with the range (0.35–0.80) of the four EST-SSR loci (SNOD1, SNOD 3, SNOD5, and SNOD8) which have been used in previously published *P. nodorum* population studies (Stukenbrock et al., 2006; Supplementary Table S3). An average of 12.65 alleles were observed for all 20 SSR loci and two loci (SNO301 and SNO1301) had notably high genetic diversity (H_exp_ > 0.90 and λ > 0.90) (Supplementary Table S3). The average Nei’s genetic diversity for all markers was 0.69 (Supplementary Table S3). The genetic diversity H_exp_ and Simpson’s index (λ) of isolates collected from each region were similar, which ranged from 0.67 to 0.70, and from 0.95 to 0.99, respectively (Table 1).

**TABLE 1:** The clonal fraction, the linkage equilibrium test results and mating idiomorphs of Norwegian *P. nodorum* isolate collection from different regions.

**Population**	**No. of isolates**	**Clone corrected**	**Clonal fraction**	**I_A_**	**p.I_A_**	**r_d_**	**p.r_d_**	**Ratio MAT**	**χ^2^**	**p.χ^2^**	**λ^a^**	**H_exp_^b^**
Hedmark	53	53	0	−0.16	0.98	−0.01	0.98	30:23	0.92	0.33	0.98	0.67
Main	92	92	0	0.03	0.30	0.002	0.30	55:37	4.26	0.06	0.99	0.70
Trøndelag	20	20	0	−0.16	1.00	−0.008	1.00	11:9	0.2	0.65	0.95	0.70
Whole population	165	164	0.01	−0.02	0.85	−0.0001	0.85	96:69	4.42*	0.04	0.99	0.69

“Main” includes three counties in the major wheat growing region (Akershus, Vestfold, and Østfold). *P* < 0.05*, I_*A*_, the index of association (Brown et al., 1980); p.I_*A*_, *p*-values of I_*A*_; r_*d*_, the standard index of association; p. r_*d*_, *p*-values of r_*d*_, χ^2^, Chi-square value for deviation from the 1:1 mating idiomorphs ratio; p. χ^2^, *p*-value for chi-square test, a: Simpson’s index (λ) (Simpson, 1949) b: unbiased gene diversity (H_*exp*_) (Nei, 1973).

### Test for Random Mating

Polymerase chain reaction amplification for mating type idiomorphs were successful for all 174 tested isolates. Mating type ratios for isolates from each of the three tested regions did not significantly deviate from the expected 1:1 ratio (Table 1). However, the mating type distribution for the whole Norwegian isolate collection was skewed with ratio MAT1-1: MAT 1-2 (96: 69) (*p* < 0.05). The test of index of association (I_A_) and standard index of association (r_d_) estimated the linkage equilibrium in the Norwegian *P. nodorum* population. From Table 1, no significant deviation was observed from the null-hypothesis of no associations between loci, indicating that the Norwegian *P. nodorum* population undergoes random mating.

### SnTox Gene Profile and Allele Frequency

**FIGURE 2:**
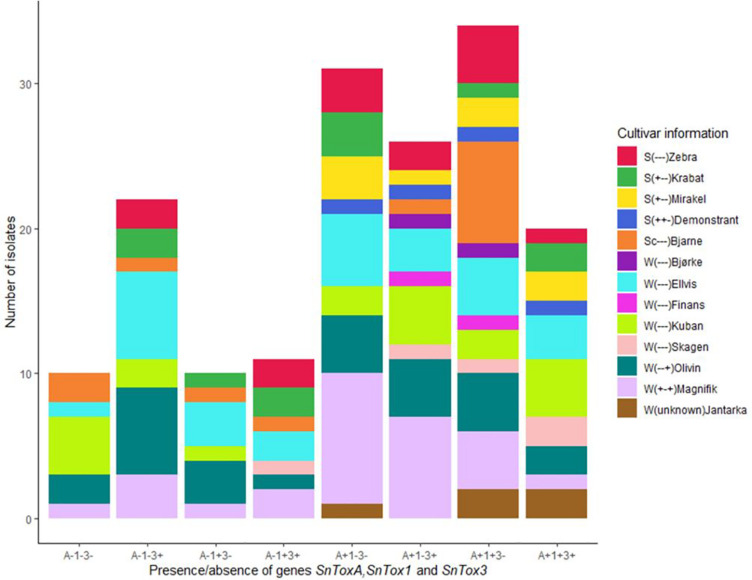
Distribution of multi-effector genotypes in the Norwegian *P. nodorum* isolate collection (*N* = 165). The wheat cultivar from which the *P. nodorum* isolates were identified are color coded. S, spring wheat; W, winter wheat. Cultivar sensitivities to the *P. nodorum* effectors ToxA, Tox1, and Tox3 are indicated in parentheses (“+”: sensitive, “–”: insensitive).

Polymerase chain reaction screening for presence/absence of the three known NE genes *SnToxA*, *SnTox1*, and *SnTox3* revealed that the Norwegian *P. nodorum* population had relatively high *SnToxA* frequency (67.9%) compared to the other two NE genes *SnTox1* (46.1%) and *SnTox3* (47.9%) (Table 2). As shown in Figure 2, genotype A+1+3- with presence of *SnToxA* and *SnTox1* but absence of *SnTox3* (*N* = 34) was the dominating multi-effector genotype and was identified in *P. nodorum* isolated from all 13 cultivars sampled in this study. The second most abundant genotype was A+1−3− (*N* = 31), and large proportions of this multi-effector genotype were isolated from the winter wheat cultivar Magnifik (Figure 2). Ten isolates in our collection did not possess any of the three known *SnTox* genes and were isolated from five different cultivars (Figure 2).

**TABLE 2:** Frequencies of the three known effectors in Norway and chi-square test for *SnTox* gene frequencies in Norway compared with the frequencies in Europe.

**Population**	***SnToxA***	***SnTox1***	***SnTox3***
Europe (McDonald et al., 2013)	12%	89%	67%
Hedmark (*N* = 53)	39(73.6%)***	24(45.3%)***	25(47.2%)**
Main (*N* = 92)	60(65.2%)***	45(48.9%)***	48(52.2%)**
Trøndelag (*N* = 20)	13(65.0%)***	7(35.0%)***	6(30.0%)***
All population (*N* = 165)	112(67.9%)***	76(46.1%)***	79(47.9%)***

*** *p* < 0.0001, ** *p* < 0.01.

A subset of 160 isolates (excluding isolates collected from the wheat variety Jantarka) were tested for the association between *SnTox* gene frequencies and the sampling cultivars (Supplementary Table S4). Chi-square test showed that *SnToxA*, *SnTox1*, or *SnTox3* frequencies in *P. nodorum* isolates were independent from the wheat cultivars from which isolates were collected (Supplementary Table S5). Additionally, Chi-square tests were carried out to test the association between *SnToxA*, *SnTox1*, or *SnTox3* frequencies with mating type, sampling location, wheat types and cultivar NE sensitivities. Except for *SnTox3*, which was associated with mating types, no significant association was observed in the Chi-square tests of the remaining *SnTox* genes (Supplementary Table S5).

### Population Structure of Norwegian *P. nodorum* Isolates

**FIGURE 3:**
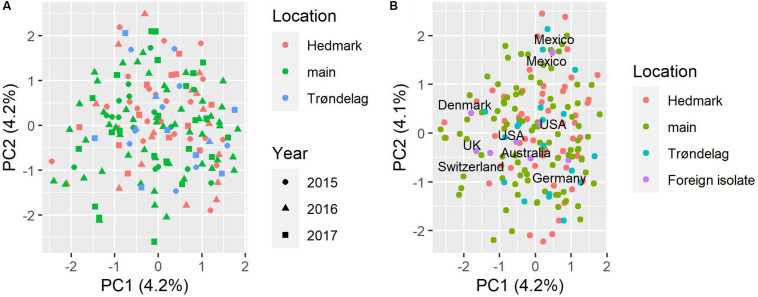
PCA analysis of population structure in *P. nodorum* isolates. **(A)** PCA scatter plot based on Norwegian isolates, sampling locations are coded by color, the year of collection is coded by shape. **(B)** PCA scatter plot based on whole isolate collection including nine isolates from outside of Norway, sampling locations in Norway are coded by color, country names of foreign isolates are indicated in the plot.

Based on the STRUCTURE analysis results, the DeltaK method indicated two genetic subpopulations (clusters) in the Norwegian *P. nodorum* collection (*K* = 2) (Supplementary Figure S2A). However, no geographical division of subpopulations was observed between the three regions investigated, with roughly equal proportions of isolates from each location assigned to the two K groups (Supplementary Figure S2C). PCA analysis for the Norwegian isolate collection and the whole collection including foreign isolates were done separately. Supplementary Figure S2B showed the PCA scatter plot of Norwegian *P. nodorum* isolates which were color coded by the two STRUCTURE genetic subpopulations, with no obvious separation of K within the PCA space observed. In addition, for the Norwegian isolates, no clear evidence of population subdivision by either location or year of collection was found (Figure 3A). Both PC1 and PC2 only explained 4.2% of the variance, which is probably due to the high genetic diversity in the Norwegian *P. nodorum* collection. In addition, PCA analysis of the whole collection including foreign isolates indicated that the two Mexican isolates clustered together and were separated from the other foreign (non-Norwegian) isolates along PC2 (Figure 3B). The Norwegian *P. nodorum* isolates showed quite high diversity and could not be differentiated from foreign isolates (Figure 3B). Population differentiation by mating type or by multi-effector genotypes in the Norwegian isolates was also tested by PCA (Supplementary Figures S3A,B), but no clear pattern of clusters could be detected.

Similar to the PCA results, the snapclust method based on analysis of Akaike information content also estimated that the most likely number of genetic subpopulations in the Norwegian *P. nodorum* collection was 1 (Supplementary Figure S4), as the lowest Akaike information criterion (AIC) value was 1 and the value increased when increasing the number of clusters estimated. The result did not change when adding all foreign isolates for the same snapclust analysis, indicating that there were no significant differences between Norwegian *P. nodorum* isolates and the foreign isolates included in our collection.

**FIGURE 4:**
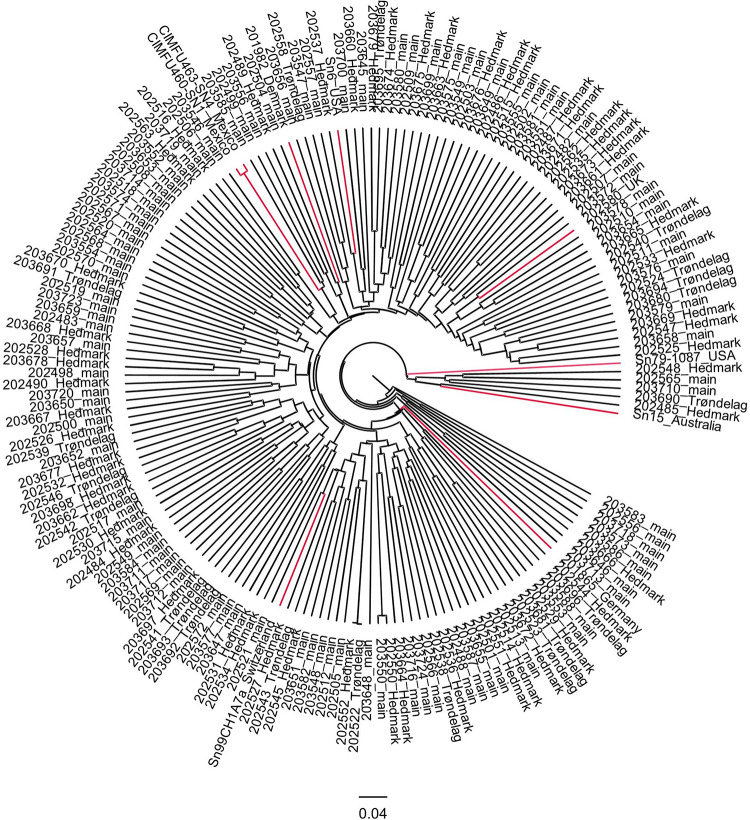
UPGMA tree of 165 Norwegian and 9 foreign *P. nodorum* isolates. Isolates are labeled by the Isolate ID and region of collection. Foreign isolates are marked in red.

According to the UPGMA tree, only isolate Sn79-1087 from the United States formed a distinct branch. All other foreign isolates were connected to Norwegian isolates by medium to long branches (Figure 4). Two Mexican isolates were clustered closely together in the UPGMA tree, which corresponded to the results from PCA (Figure 3). Most *P. nodorum* isolates were connected by long branches (Figure 4), revealing the existence of high genetic diversity in the Norwegian *P. nodorum* population. Analysis of AMOVA also revealed that high genetic variance existed within predicted populations rather than between populations (Supplementary Table S6), which was expected as the predicted subpopulations were not significantly differentiated.

## Discussion

Table 1 showed that mating type ratio of the whole country deviated significantly (*p* < 0.05) from 1:1. However, the ratio deviation for each location was not significantly different from 1:1, even though more MAT1-1 types were observed than MAT1-2 types in all locations (Table 1). It is possible that this might be caused by type I error and false rejection of the null hypothesis, since the *p*-value was just 0.04. However, there are other possibilities which could explain this result. For example, according to Sommerhalder et al. (2006) when seedborne inoculum played the major role for *P. nodorum* primary inoculation, it might lead to a skewed ratio of mating types with rare sexual reproduction. In addition, asexual inoculum coming from wheat debris might also serve as primary inoculum due to the reduced tillage practices (Mehra et al., 2015). The ratio of mating types deviated significantly from the 1:1 ratio when considering the nationwide Norwegian *P. nodorum* population (Table 1). However, the Norwegian *P. nodorum* population showed high genetic diversity, low clonal fraction, and the mating type ratios were not significantly different from 1:1 in any tested location indicating that the Norwegian *P. nodorum* population undergoes regular sexual reproduction. Moreover, index of association (I_A_) and standard index of association (r_d_) results suggests that the Norwegian *P. nodorum* population underwent random mating (Table 1). Ascospores have also been found regularly in southeast Norway by trapping them with Burkhard spore traps (Ficke et al., 2016). We hypothesize that the major *P. nodorum* primary inoculum in Norway are ascospores, however, combined with considerable amount of seedborne and/or residue-borne inoculum. The regular sexual recombination contributes to the high genetic diversity. Therefore, no clear subdivision of the pathogen population was observed by either PCA or the snapclust method. In addition, the deltaK approach used to estimate the best value of K from STRUCTURE analysis, can only be applied when the clusters in one population are equal to or more than 2, which overestimate the structure when *K* = 1 was the potential solution (Janes et al., 2017). Therefore, the *K* = 2 estimated by this method is likely an artifact, since there was no clear separation of isolates grouped to cluster 1 and cluster 2 (Supplementary Figure S2B). In addition, statistical analysis of molecular variance (AMOVA) also supported our conclusion that no genetic subdivision of the Norwegian isolate collection is present (Supplementary Table S6).

*Septoria tritici* blotch disease is the major problem on winter wheat in Europe, as *Z. tritici* ascospores are surviving on stubble between crops (Fones and Gurr, 2015). Large volumes of *Z. tritici* ascospores are produced around crop harvesting time in the summer and autumn sown winter wheat provides a bridge for *Z. tritici* winter survival (Eriksen and Munk, 2003). Countries neighboring Norway such as Denmark and Sweden grow winter wheat at quite large scales compared to spring wheat (Statistics Sweden, 2018; Statistics Denmark, 2019). However, in Norway typically, more spring wheat is planted than winter wheat due to difficulties in sowing during the typically wet Norwegian autumns (Statistics Norway, 2018). We hypothesize that STB is not the major leaf blotch pathogen in Norwegian spring wheat due to the lack of sufficient developing time for the long latent period which *Z. tritici* needs in comparison to *P. nodorum* (Cunfer, 1999). This could also explain the relatively higher occurrence of *Z. tritici* on winter wheat than spring wheat in Norway. Moreover, *P. nodorum* would compete with *Z. tritici* on winter wheat, but will encounter less competition on spring wheat in Norway. Because of this we first expected that the pathogen populations infecting Norwegian spring wheat might be different from the populations on winter wheat. However, the PCA scatter plot (Supplementary Figure S3D) showed that this was not the case, as no clear differentiation of pathogen populations was observed by wheat type, suggesting that there is effectively only one large and diverse *P. nodorum* population infecting both winter wheat and spring wheat in Norway. A recent study by Richards et al. (2019) found two different *P. nodorum* populations in the United States corresponding to spring wheat and winter wheat growing regions, respectively, which was likely due to distinct selection pressure caused by the cultivars grown in each region. However, both spring wheat and winter wheat are grown in all wheat growing regions in Norway (and even in the same field but in different years). Therefore, the selection pressure on *P. nodorum* in Norway is likely multidirectional and non-constant, which might also contribute to the high genetic diversity in the pathogen population. High genetic variability within *P. nodorum* populations has also been reported by other population genetic studies in Australia, Sweden, and even the global collection (Murphy et al., 2000; Stukenbrock et al., 2006; Blixt et al., 2008) indicating that this is a common feature in *P. nodorum*.

Ruud et al. (2018) studied the *SnTox* gene frequencies of 62 Norwegian *P. nodorum* isolates, collected from spring wheat during 2012 to 2014, and the allele frequencies for *SnToxA*, *SnTox1*, and *SnTox3* were 0.69, 0.53, and 0.76, respectively. Table 3 shows the *SnTox* gene allele frequencies in our collection compared to the *P. nodorum* collection by Ruud et al. (2018) where significantly lower *SnTox3* frequencies were observed in our collection (*p* < 0.001). Significant differences were also detected when comparing *SnTox3* allele frequency in Ruud et al. (2018) with subsets of isolates collected from either spring wheat or winter wheat in our study. One possible explanation could be the relatively small sample size of the Ruud et al. (2018) collection. Those isolates might not be as representative of the highly diverse *P. nodorum* pathogen population in Norway as the isolates used in our current study. In addition, the *SnTox* gene allele frequencies in a population may also change due to adaptation to the host NE sensitivities. The *SnToxA* frequency in our Norwegian *P. nodorum* isolate collection (67.9%) was significantly higher than the European level (12%), while the *SnTox1* (46.1%) and *SnTox3* (47.9%) frequencies were significantly lower than the European levels (McDonald et al., 2013; Table 2). Similar high *SnToxA* frequency was also observed in the *P. nodorum* isolate collection by Ruud et al. (2018). Furthermore, Ruud et al. (2018) found that large proportions (46.5%) of Norwegian spring wheat cultivars and breeding lines carry the ToxA sensitivity allele, *Tsn1*. This is in contrast to a previous study of north-west European wheat varieties, which found high ToxA sensitivity to be present in just ∼10% of varieties (Downie et al., 2018). We sampled isolates from 13 commercial wheat cultivars grown in Norway, which collectively covered more than 95% of the seed sales in the growing seasons (2015–2017) (Åssveen et al., 2018). Of these 13 cultivars, 30% were sensitive to ToxA, much higher than the proportion sensitive to either Tox1 (7.6%) or Tox3 (15.3%) (Supplementary Table S4). In addition, we found that three of the four ToxA sensitive cultivars were spring wheat. Ruud et al. (2018) found that field SNB disease level in Norway was significantly correlated with ToxA sensitivity in cultivars. While *SnTox1* has previously been found to be the most prevalent effector gene in the wider European *P. nodorum* population (89%) (McDonald et al., 2013) in Norway we found significantly lower *SnTox1* allele frequencies (46.1%). This is possibly due to the low selection pressure for it in Norway, as few cultivars grown in Norway are Tox1 sensitive (Ruud et al., 2018). Varieties sensitive to ToxA but insensitive to Tox3 have been shown to have the highest SNB disease severity in Norway (Ruud et al., 2018). Our *SnTox* gene profile results showed that the most abundant multi-effector genotype in our Norwegian isolate collection was A+1+ 3−, and the second largest genotype was A+1−3− (Figure 2). This A+(1±)3− genotype accounted for nearly 40% of the total Norwegian *P. nodorum* collection, which suggests that the NE profile of the pathogen population is under selection by corresponding NE sensitivities of the host.

**TABLE 3:** Frequencies of the three known effectors in Norway and chi-square test for *SnTox* frequency in Norway compared with another Norwegian *P. nodorum* collection.

**Number of isolates**	***SnToxA***	***SnTox1***	***SnTox3***
Ruud et al. (2018) collection (*N* = 62)	43(69%)	33(53%)	47(76%)
Spring wheat (*N* = 51)	37(72.5%)	29(56.9%)	21(41.2%)***
Winter wheat (*N* = 114)	75(65.8%)	47(41.2%)	58(50.9%)**
Total (*N* = 165)	112(67.9%)	76(46.1%)	79(47.9%)***

The number of isolates included were labeled in parenthesis. *** *p* < 0.001, ** *p* < 0.01.

The *SnToxA* frequency in *P. nodorum* isolates collected from ToxA sensitive cultivars was expected to be higher compared to the ToxA insensitive cultivars, as Richards et al. (2019) found low *SnToxA* frequency in the *P. nodorum* population where *Tsn1* is rare in the region, indicating there might be a fitness cost to carrying the *SnToxA* gene. However, in our study, no significant difference was detected in *SnToxA* frequencies between isolates collected from ToxA sensitive and insensitive cultivars and the population was not clustered by cultivar sensitivity to ToxA (Supplementary Figure S3C). We hypothesize that because of the inconsistent and multilateral selection pressure caused by growing cultivars with different NE sensitivities in Norway, carrying *SnToxA* still gives higher levels of competitive ability regardless of the potential fitness cost. In addition, a recent study from Peters et al. (2019) revealed that *P. nodorum* isolates might not express all the necrotrophic effector genes they possess simultaneously. Depending on the host NE sensitivities, *P. nodorum* isolates would “adjust” expression profile of the effectors to the host sensitivity (Peters et al., 2019). This may therefore modulate any cost of carrying a specific effector gene.

In summary, *P. nodorum* is considered as a pathogen with relatively high evolutionary potential and high expected risk (McDonald and Linde, 2002) which would facilitate rapid adaptation to and breaking of host qualitative genetic resistance. Because of its ability of rapid local adaptation, the high frequency of the *Tsn1* allele existing in Norwegian spring wheat cultivars has helped to maintain the large *P. nodorum* population in Norway. Moreover, different selection pressures caused by cultivars with different NE sensitivities in spring and winter wheat may contribute to the high genetic diversity of the Norwegian *P. nodorum* population. Our results also suggest that eliminating the *Tsn1* allele in Norwegian spring wheat cultivars might be a possible way to reduce SNB severity, as this approach was successfully applied in Australia (Vleeshouwers and Oliver, 2014). However, more research on this sophisticated *P. nodorum* -wheat pathosystem is still needed, as the diversified *P. nodorum* population in Norway harbors many other NE genes (Lin et al., 2020) which may enable rapid adaptation to new susceptibility loci once the *Tsn1* allele is removed from wheat cultivars. In addition, a genome wide association study (GWAS) of SNB field resistance (Ruud et al., 2019) showed that wheat genetic resistance to SNB were quantitative while sensitivities to known NEs could only explain small proportions of the phenotypic variation.

## Data Availability Statement

The datasets generated for this study can be found as Supplementary Table S7.

## Author Contributions

MiL, AF, and MoL collected leaf samples. MiL conducted the experiments, analyzed the data, and drafted the manuscript. MoL and JC gained project funding. MoL, AF, and JC supervised the research. All authors revised and approved the manuscript.

## Conflict of Interest

The authors declare that the research was conducted in the absence of any commercial or financial relationships that could be construed as a potential conflict of interest.

## Supplementary Material

FIGURE S1 The genotype-accumulation curve for 174 *P. nodorum* isolates genotyped by 20 SSR markers. The horizontal axis indicates the number of SSR loci randomly sampled up to (n-1) loci, and the vertical axis represents the number of multilocus genotypes. The red-dashed line represents the total number of multilocus genotype observed in the collection.

FIGURE S2 Genetic structure analysis of the Norwegian *P. nodorum* population. **(A)**, DeltaK plot, indicating *K* = 2 or lower as the best fit for the Norwegian *P. nodorum* population. **(B)**, PCA scatter plot of Norwegian *P. nodorum* isolates color coded by the predicted clusters (genetic substructure) from STRUCTURE results. **(C)**, Estimated genetic substructure across all 165 Norwegian isolates using STRUCTURE (*K* = 2), vertical bars indicate the probability of each isolate being assigned to the two substructure groups.

FIGURE S3 PCA scatter plot of Norwegian *P. nodorum* isolates according to: **(A)** NE profiles, **(B)** mating type, **(C)** host cultivars sensitivity to ToxA, **(D)** wheat type of host cultivar (S, spring wheat; W, winter wheat).

FIGURE S4 The Akaike information criterion (AIC) value of different number of clusters (K) considered in Norwegian *P. nodorum* isolate collection estimated by “snapclust.”

TABLE S1 Newly developed SSR primer sequences.

TABLE S2 Field sampling information.

TABLE S3 Number of SSR alleles detected, Simpson’s index, Nei’s gene diversity H_exp_ and evenness for each SSR marker. a: Simpson’s index (λ) (Simpson, 1949), b: unbiased gene diversity (H_exp_) (Nei, 1973).

TABLE S4 Wheat cultivar type and sensitivity to the three known *P. nodorum* NEs: ToxA, Tox1, and Tox3.

TABLE S5 Chi-square test for independence of *SnTox* frequency and cultivars where isolates were collected from. NE, necrotrophic effector.

TABLE S6 Analysis of molecular variance (AMOVA). Df, degree of freedom.

TABLE S7 SSR marker data, sampling information and genotyping results on all isolates used in this study.
