# Supplementary figures and images for: Genetic Structure of the Norwegian Parastagonospora nodorum Population
Source: Front Microbiol. 2020 Jun 16;11:1280. doi: 10.3389/fmicb.2020.01280 (PMC7309014; doi:10.3389/fmicb.2020.01280)

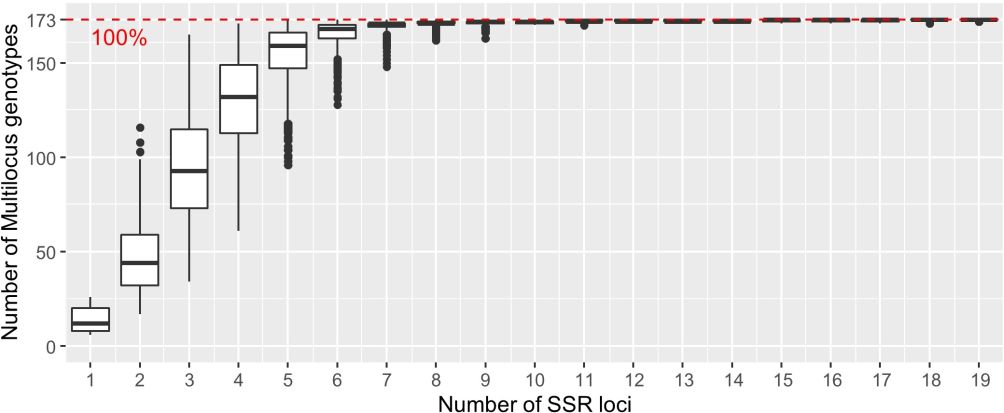

Supplement: FIGURE S1 — The genotype-accumulation curve for 174 P. nodorum isolates genotyped by 20 SSR markers. The horizontal axis indicates the number of SSR loci randomly sampled up to (n-1) loci, and the vertical axis represents the number of multilocus genotypes. The red-dashed line represents the total number of multilocus genotype observed in the collection. [file Data_Sheet_2.zip › Figure S1.JPEG]

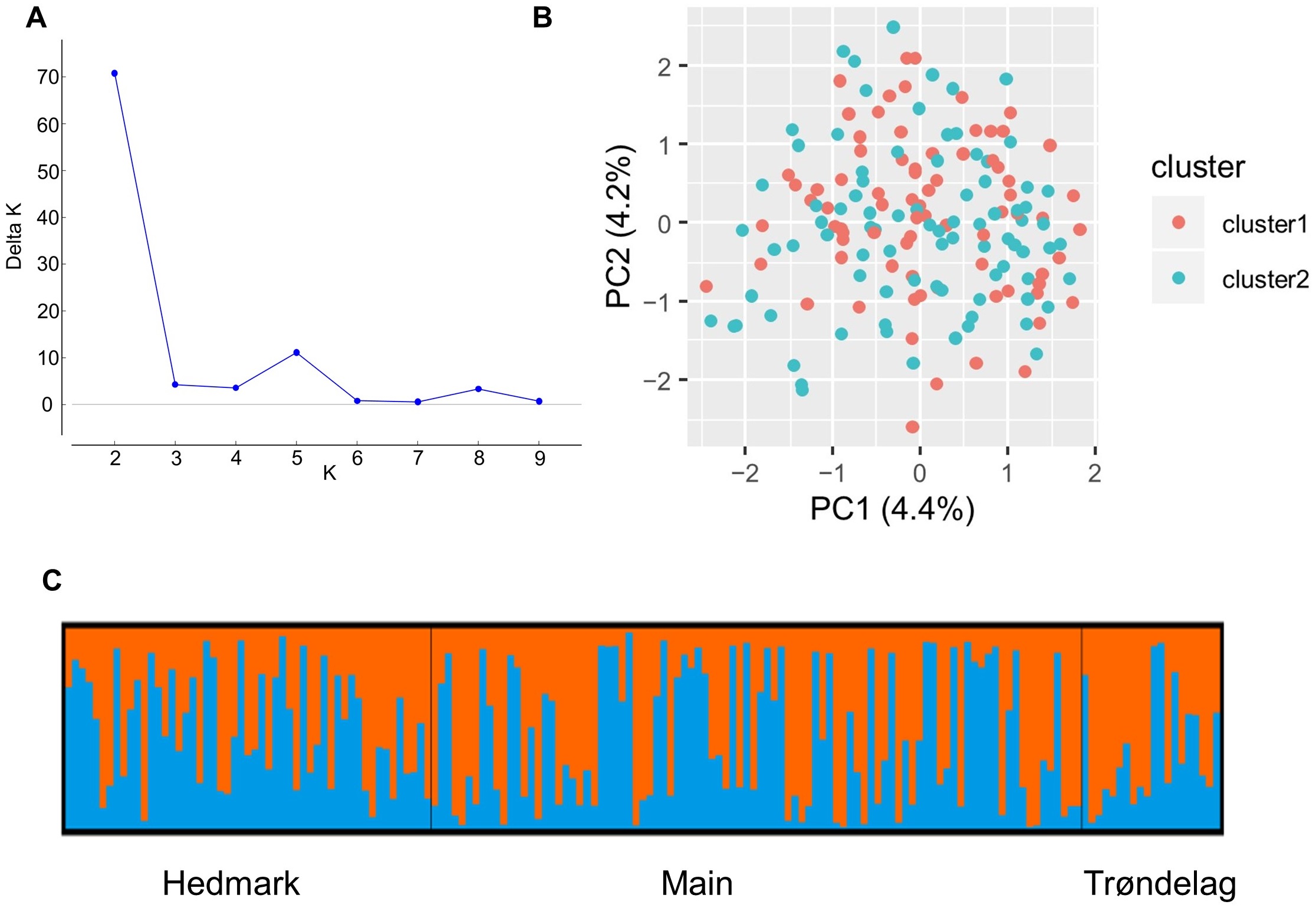

Supplement: FIGURE S1 — The genotype-accumulation curve for 174 P. nodorum isolates genotyped by 20 SSR markers. The horizontal axis indicates the number of SSR loci randomly sampled up to (n-1) loci, and the vertical axis represents the number of multilocus genotypes. The red-dashed line represents the total number of multilocus genotype observed in the collection. [file Data_Sheet_2.zip › Figure S2.JPEG]

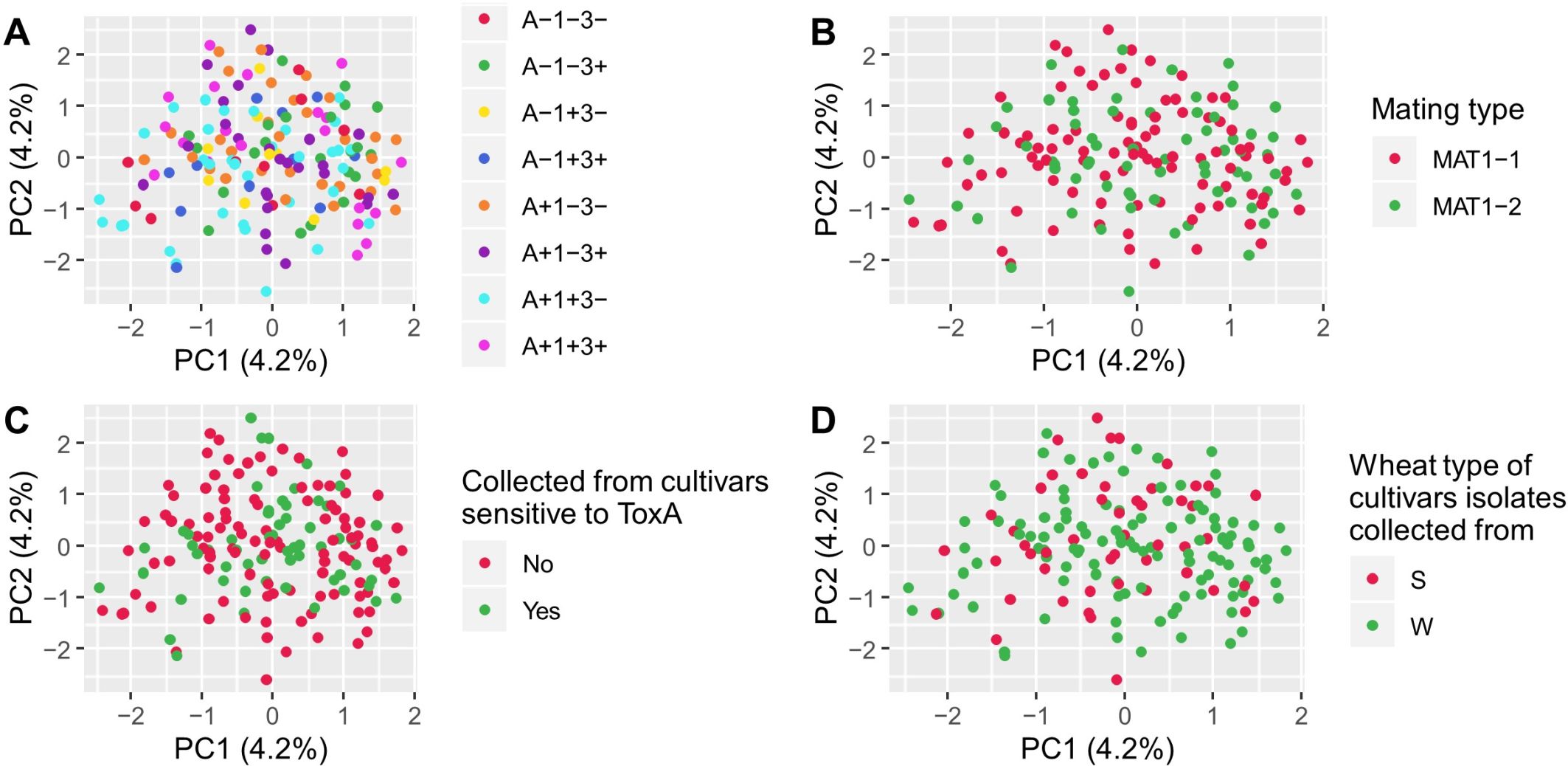

Supplement: FIGURE S1 — The genotype-accumulation curve for 174 P. nodorum isolates genotyped by 20 SSR markers. The horizontal axis indicates the number of SSR loci randomly sampled up to (n-1) loci, and the vertical axis represents the number of multilocus genotypes. The red-dashed line represents the total number of multilocus genotype observed in the collection. [file Data_Sheet_2.zip › Figure S3.JPEG]

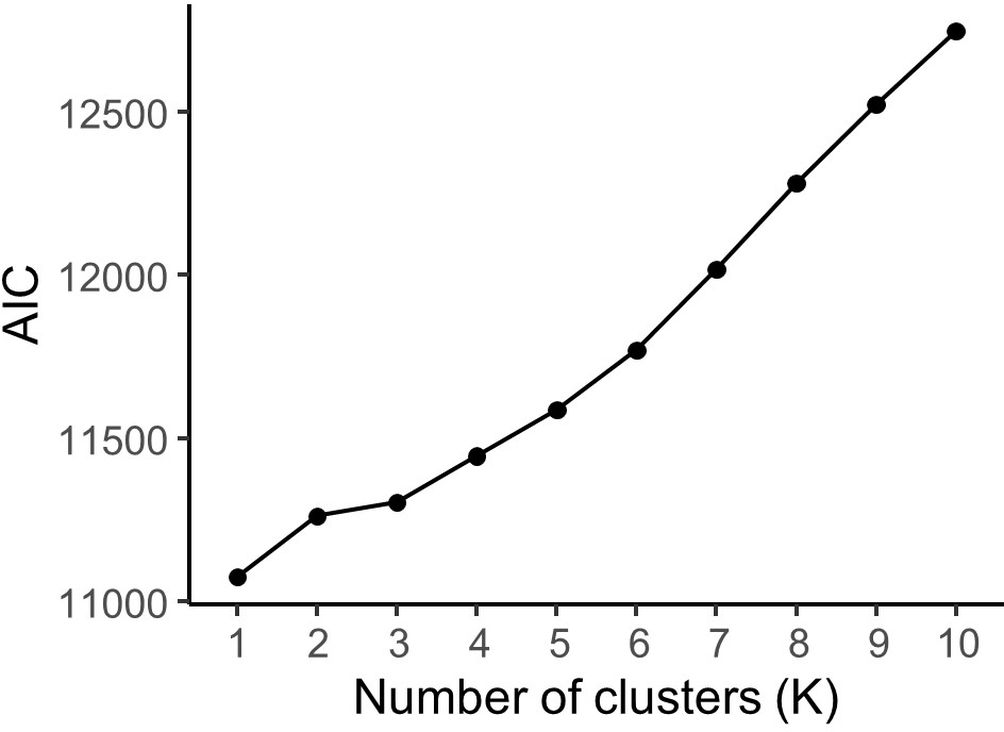

Supplement: FIGURE S1 — The genotype-accumulation curve for 174 P. nodorum isolates genotyped by 20 SSR markers. The horizontal axis indicates the number of SSR loci randomly sampled up to (n-1) loci, and the vertical axis represents the number of multilocus genotypes. The red-dashed line represents the total number of multilocus genotype observed in the collection. [file Data_Sheet_2.zip › Figure S4.JPEG]
