# Supplementary material for: Genetic Structure of the Norwegian Parastagonospora nodorum Population
Source: Front Microbiol. 2020 Jun 16;11:1280. doi: 10.3389/fmicb.2020.01280 (PMC7309014; doi:10.3389/fmicb.2020.01280)
Supplement: TABLE S1 — Newly developed SSR primer sequences. [file Data_Sheet_1.zip › Table S1-S6.DOCX]

Supplementary Material

# Supplementary Tables

Table S1 Newly developed SSR primer sequences

| SSR Name | Repeat motif | Labelling dye | Primer left (5’ to 3’) | Primer right (5’ to 3’) | PCR product size range (bp) |
| --- | --- | --- | --- | --- | --- |
| SNO101 | ACC | PET | TCGGTCAGAAGCATAACGGT | TCGCGATGACTACGGCTC | 155-179 |
| SNO301 | TC | NED | CAGGGCATCATTTCGTAACGT | GGGAGGTGTGTCTGGTACTT | 114-174 |
| SNO402 | TTG | FAM | GAGAGCGTTGTTGTTGCCA | GCCAAGGTCAGAACAACGG | 113-158 |
| SNO501 | GTT | VIC | CTGGGTGTTCTGCTCGTTG | ACTCTACATCACTCATCATGGAC | 112-130 |
| SNO702 | ACC | PET | CAAACGAGCCTCATACGACC | TATAAGGTGGTTCTCGGCGG | 123-144 |
| SNO801 | GCA | NED | ACAATCACATGCCATCCCTG | ACTATCTGGTCGAGCGTCTG | 108-153 |
| SNO802 | TCTAC | NED | TTCTATCAGTCGCGGATCAC | TGGAGATAGCGAGAATGGCA | 95-225 |
| SNO901 | TGT | FAM | CTGCTTCTCGGTCGCCAT | TCGTCTTCCATCTGTCCACG | 188-257 |
| SNO1001 | TG | PET | GTAGACCAGCTGCGGAAATG | CAAGCTACTACCCACTTTACAGG | 149-229 |
| SNO1002 | TGG | VIC | GTGTTGGTATTGTAACGTGTGC | CTACCCCAGCTACACCACG | 123-144 |
| SNO1301 | TCT | FAM | TGGCGAACAGTAGAGGATCC | TTCGCCAATGATGTCACGTC | 185-266 |
| SNO1302 | GGCGTA | NED | TCTTGGATGGTGTGAAGCCT | CATCTTTACGGGCGCATTGA | 87-159 |
| SNO1801 | TCA | FAM | TGCACGCCTCTTCAAATAGC | GCCACCCTATTTCTCTTCTGG | 198-351 |
| SNO1802 | CA | VIC | GCTGCACTTCATACTCAGATAGC | GAGGGTGTCGATTGAGGTCA | 116-202 |
| SNO3001 | GAA | NED | GCTTCTCCGACAACACTGC | CGATCTCAGTCGGCAGTACT | 90-240 |
| SNO3701 | GAA | PET | CAAACACAACACGCGCGG | AGGCAATGTATACCCGCCAT | 133-145 |

Table S2 Field sampling information

| Region | Year collected | Number of isolates collected | Wheat cultivar(s) | Wheat type |
| --- | --- | --- | --- | --- |
| Trøndelag | 2015 | 9 | Magnifik, Olivin | Winter wheat |
| Trøndelag | 2016 | 4 | Magnifik,Bjarne, Zebra | Winter wheat, Spring wheat |
| Trøndelag | 2017 | 7 | Magnifik, Ellvis, Bjarne, Zebra,Mirakel, | Winter wheat,  Spring wheat |
| Hedmark | 2015 | 22 | Ellvis, Kuban, Magnifik, Olivin | Winter wheat |
| Hedmark | 2016 | 18 | Ellvis, Kuban, Magnifik, Olivin, Jantarka, Bjarne, Skagen, Demonstrant, Krabat, Zebra | Winter wheat,  Spring wheat |
| Hedmark | 2017 | 13 | Ellvis, Kuban, Magnifik, Olivin, Bjarne, Krabat, Mirakel, Zebra | Winter wheat,  Spring wheat |
| Akershus, Østfold, Vestfold | 2015 | 17 | Ellvis, Kuban, Magnifik, Olivin | Winter wheat |
| Akershus, Østfold, Vestfold | 2016 | 52 | Bjoerke, Ellvis, Finans, Jantarka, Kuban, Magnifik, Olivin, Skagen, Bjarne, Demonstrant, Krabat, Mirakel, Zebra | Winter wheat,  Spring wheat |
| Akershus, Østfold, Vestfold | 2017 | 23 | Ellvis, Kuban, Magnifik, Olivin, Bjarne, Krabat, Mirakel, Zebra | Winter wheat,  Spring wheat |
| Other countries (Denmark, Germany, Switzerland, UK, USA, Mexico, and Australia) |  | 9 |  |  |

Table S3 Number of SSR alleles detected, Simpson`s index, Nei`s gene diversity H_exp_ and evenness for each SSR marker

| SSR | Alleles | λ^a^ | H_exp_^b^ | Evenness |
| --- | --- | --- | --- | --- |
| SNOD1 | 9 | 0.37 | 0.37 | 0.41 |
| SNOD3 | 6 | 0.35 | 0.35 | 0.47 |
| SNOD5 | 12 | 0.79 | 0.80 | 0.69 |
| SNOD8 | 5 | 0.40 | 0.40 | 0.64 |
| SNO101 | 7 | 0.63 | 0.64 | 0.69 |
| SNO301 | 21 | 0.90 | 0.90 | 0.77 |
| SNO402 | 12 | 0.80 | 0.80 | 0.75 |
| SNO501 | 7 | 0.56 | 0.56 | 0.68 |
| SNO702 | 8 | 0.81 | 0.81 | 0.84 |
| SNO801 | 15 | 0.88 | 0.89 | 0.81 |
| SNO802 | 11 | 0.56 | 0.56 | 0.51 |
| SNO901 | 10 | 0.79 | 0.80 | 0.79 |
| SNO1001 | 15 | 0.79 | 0.80 | 0.62 |
| SNO1002 | 8 | 0.63 | 0.63 | 0.62 |
| SNO1301 | 24 | 0.92 | 0.93 | 0.77 |
| SNO1302 | 10 | 0.67 | 0.67 | 0.67 |
| SNO1801 | 23 | 0.87 | 0.88 | 0.69 |
| SNO1802 | 20 | 0.85 | 0.86 | 0.63 |
| SNO3001 | 25 | 0.47 | 0.47 | 0.29 |
| SNO3701 | 5 | 0.71 | 0.72 | 0.80 |
| Mean | 12.65 | 0.69 | 0.69 | 0.66 |

a: Simpson`s index (λ) (Simpson, 1949),

b: unbiased gene diversity (H_exp_) (Nei, 1973)

Table S4 Wheat cultivar type and sensitivity to the three known *P. nodorum* NEs: ToxA, Tox1 and Tox3

| Cultivar name | No. of isolates collected from | Wheat type | Cultivar sensitivity to | | |
| --- | --- | --- | --- | --- | --- |
|  |  |  | ToxA | Tox1 | Tox3 |
| Bjarne | 14 | S | - | - | - |
| Demonstrant | 4 | S | + | + | - |
| Krabat | 11 | S | + | - | - |
| Mirakel | 8 | S | + | - | - |
| Zebra | 14 | S | - | - | - |
| Bjørke | 2 | W | - | - | - |
| Ellvis | 27 | W | - | - | - |
| Finans | 2 | W | - | - | - |
| Jantarka | 5 | W | unknown | unknown | unknown |
| Kuban | 19 | W | - | - | - |
| Magnifik | 28 | W | + | - | + |
| Olivin | 26 | W | - | - | + |
| Skagen | 5 | W | - | - | - |
| Total |  |  | 4 (30.1%) | 1 (7.6%) | 2 (15.3%) |

S: spring wheat, W: winter wheat, +: sensitive, -: insensitive

Table S5 Chi-square test for independence of *SnTox* frequency and cultivars where isolates were collected from. NE = necrotrophic effector

|  | Cultivar (χ^2^) | *P. nodorum* mating type (χ^2^) | Sampling location (χ^2^) | Wheat type (χ^2^) | Cultivars`NE sensitivity (χ^2^) |
| --- | --- | --- | --- | --- | --- |
| *ToxA* frequency | 16.51 | 0.01 | 1.28 | 0.61 | 5.37 |
| *Tox1* frequency | 12.69 | 1.23 | 1.91 | 3.06 | 4.82 |
| *Tox3* frequency | 6.38 | 4.58* | 2.71 | 1.01 | 0.09 |

*: p < 0.05

Table S6 Analysis of molecular variance (AMOVA). Df: degree of freedom

|  | Df | Sum of squares | Mean square | Variance | Percentage % | Phi statistics | P value |
| --- | --- | --- | --- | --- | --- | --- | --- |
| By locations | | | | | | | |
| Between regions | 2 | 30.29 | 15.15 | 0.03 | 0.21 |  |  |
| Within regions | 161 | 2223.61 | 13.81 | 13.81 | 99.79 |  |  |
| Total | 163 | 2253.91 | 13.83 | 13.84 | 100.00 | 0.002 | 0.65 |
| By cultivars | | | | | | | |
| Between Cultivars | 12 | 162.69 | 13.56 | -0.02 | -0.17 |  |  |
| Within Cultivars | 151 | 2091.22 | 13.85 | 13.85 | 100.17 |  |  |
| Total | 163 | 2253.91 | 13.83 | 13.82 | 100.00 | -0.002 | 0.66 |
| By Years | | | | | | | |
| Between years | 2 | 25.67 | 12.84 | -0.02 | -0.14 |  |  |
| Within years | 161 | 2228.23 | 13.84 | 13.84 | 100.14 |  |  |
| Total | 163 | 2253.91 | 13.83 | 13.82 | 100.00 | -0.001 | 0.73 |
| By Mating type | | | | | | | |
| Between Mating type | 1 | 13.96 | 13.96 | 0.002 | 0.01 |  |  |
| Within mating type | 162 | 2239.95 | 13.83 | 13.83 | 99.99 |  |  |
| Total | 163 | 2253.91 | 13.83 | 13.83 | 100.00 | 0.0001 | 0.44 |
| Between *SnToxA* presence and absence | | | | | | | |
| Between *SnToxA* presence and absence | 1 | 14.65 | 14.65 | 0.01 | 0.08 |  |  |
| Within *SnToxA* presence or absence group | 162 | 2239.26 | 13.82 | 13.82 | 99.92 |  |  |
| Total | 163 | 2253.91 | 13.83 | 13.83 | 100.00 | 0.0008 | 0.33 |
| Between locations and years | | | | | | | |
| Between locations | 2 | 30.29 | 15.15 | 0.07 | 0.52 | 0.005 | 0.06 |
| Between years within location | 6 | 71.51 | 11.92 | -0.12 | -0.85 | -0.009 | 0.99 |
| Within year | 155 | 2152.11 | 13.88 | 13.88 | 100.33 | -0.003 | 0.94 |
| Total | 163 | 2253.91 | 13.83 | 13.84 | 100.00 |  |  |
